# The Experience and Perceived Consequences of the 2016 Fort McMurray Fires and Evacuation

**DOI:** 10.3389/fpubh.2021.641151

**Published:** 2021-11-11

**Authors:** Laura Thériault, Geneviève Belleville, Marie-Christine Ouellet, Charles M. Morin

**Affiliations:** School of Psychology, Laval University, Québec, QC, Canada

**Keywords:** natural disaster, wildfire (bushfire), evacuation, consequences, qualitative research

## Abstract

Few studies have examined the scope of the subjective experience during and after a natural disaster. This qualitative study explored the perceptions of persons affected by the wildfires and evacuation of Fort McMurray in 2016. The objectives were to document (1) the experience of the evacuation, and (2) the biopsychosocial consequences of the wildfires as perceived by evacuees from Fort McMurray 3 months and 3 years after evacuation. This study included two data collections, one from 393 evacuees 3 months after evacuation using an online questionnaire, and the other from 31 participants (among those who participated in the 3-month evaluation) interviewed by telephone 3 years after evacuation. Eight themes describing the evacuation experience emerged from the qualitative analysis: the preparation for evacuation, the perceived traumatic nature of the evacuation, problems encountered while on the move, assistance received and provided, vulnerability conditions, presence of physical discomfort, relocation and no problem/no response. Seven categories of negative consequences emerged: material and financial loss, emotional/mental health disorders, cognitive impairments, behavioral changes, spiritual/existential reflections, social alterations, and physical conditions. Four categories of positive consequences emerged: posttraumatic growth, resilience/absence of consequences, altruism and community cohesion. This study showed a wide range of perceived consequences of fires and evacuations by Fort McMurray residents. The results highlight the importance of tailoring responses to the needs of evacuees and providing assistance to victims over a long period of time.

## Introduction

Every year in Canada, several disasters occur (earthquakes, floods, landslides, tornadoes, etc.) (1). One major disaster that occurred in Canada was a wildfire that raged in Fort McMurray, Alberta, in May 2016. At first, the fire appeared to be under control. However, it spread rapidly with the arrival of high winds on May 3rd (2). The evacuation of the whole population became mandatory when the fire crossed the Athabasca river in the northern part of the city. About an hour after the evacuation was announced, the fire reached the city and blocked one of the two main routes out of the city (3). This fire forced more than 80,000 residents to evacuate the city and burned approximately 2,400 homes and buildings (4). There were no fire-related deaths, but two people lost their lives in a car accident during the evacuation. More than a quarter of the population required the assistance of mental health professionals (4). This type of disaster can have major economic, social, physical and psychological impacts (5), which may lead to long-term consequences for victims (6, 7).

Few theoretical models can explain the full range of consequences following a disaster and, to the best of our knowledge, none of them concern natural disasters. The Psychosocial Risk Assessment and Management Framework (P-RAM) proposes a structured model to identify a wide range of psychosocial consequences potentially associated with terrorist threats and attacks in order to guide emergency planners, decision makers, and responders in their interventions (8). Many elements proposed by the P-RAM model are applicable to an analysis of post-disaster consequences. In particular, the model posits that threats or events can bring about normal and abnormal psychosocial effects. The normal effects can be psychosocial adverse effect such as changes in the behavioral, emotional, cognitive, social, physical and spiritual spheres. Abnormal effects include the development of mental health disorders [for example, depression, anxiety disorders, or post-traumatic stress disorder (PTSD) or other disorders presented in the Diagnostic and Statistical Manual of Mental Disorders (DSM-IV) nosology], as well other psychosocial issues such as burnout or family violence. In addition, positive reactions or psychosocial benefits can be observed after a disaster, such as resilience, community cohesion, protective behaviors, and post-traumatic growth.

Even if evacuations are ordered to protect populations in imminent danger (9), the evacuation in itself can be a very stressful event, as can be the relocation process, thus potentially negatively impacting psychological health (10). Studies have shown increased levels of post-traumatic symptoms (11), depression and anxiety disorders (12–15) in evacuees and persons relocated in the context of disasters. Afifi et al. (16) reported that the evacuation process increased uncertainty about personal safety, home security and the duration of relocation. Another study of evacuees from the 2014 wildfires in the Northwest Territories of Canada reported that fear, stress, and uncertainty contributed to negative outcomes (17). However, most studies of evacuations following a disaster focus primarily on the choice of whether or not to evacuate, not on the process of evacuation *per se* and consequences that ensue.

Beyond symptoms such as fear or insecurity, more severe forms of mental health issues have been the object of much scientific attention following disasters (10), categorized as abnormal effects in the P-RAM model. Our team conducted interviews three months after the Fort McMurray wildfires and found that 29,1% of the participants had a probable PTSD diagnosis, 25,5% a probable depression and 43,6%, insomnia (18). Another study showed that six months after the disaster, there was an incidence of 19,8% of generalized anxiety disorder (GAD) among participants from Fort McMurray (19). Participants presenting this diagnosis were also at greater risk of substance abuse. Even 18 months after the wildfires, one study found that 13,6% of participants met criteria for PTSD, 24,8% met criteria for major depressive disorder (MDD) and 18% met criteria GAD (20).

In terms of physical effects, smoke inhalation during wildfires may cause temporary, progressive or permanent breathing problems (21) and lead to respiratory and vascular diseases (22). Some elements contained in the smoke from wildfires may even have carcinogenic properties (23). Nevertheless, the literature remains undeveloped with respect to the consequences on physical health following a wildfire and even more so outside of the United States (24). A team went to Fort McMurray 14 months after the fires to investigate the presence of toxic substances (e.g., arsenic, heavy metals) in the community. They found that the levels of these substances were not higher than in unaffected homes elsewhere in Canada (25). However, even when a danger is not objectively present, the affected population could still fear or perceive physical consequences following a disaster.

Other consequences of disasters are economic and can lead to significant financial stress for the community (24). The Fort McMurray wildfires were considered Canada's most expensive natural disaster at the time, with a $3.58 billion cost (26). Very few studies have focused on the perceived financial aspect of wildfires. Kulig et al. (27) administered 52 interviews to document several potential economic consequences on wildfire evacuees. In the qualitative component of this mixed-method study, participants reported financial problems due to the loss of their homes, insurance problems, job loss or financial burden due to relocation (27). Personal and financial losses due to disasters have been shown to be associated with increased psychopathology including depression, anxiety, somatic complaints, stress and PTSD (24, 28–30).

Natural disasters can also have negative social impacts, often because the needs for support outweigh the actual support available in the community. In addition, relocation and job losses deprive victims of opportunities for social support, as they no longer have access to the people who usually provide them with this support (31). The deterioration in social support networks can represent a risk factor for developing mental health problems following a natural disaster (32).

**Figure 1:**
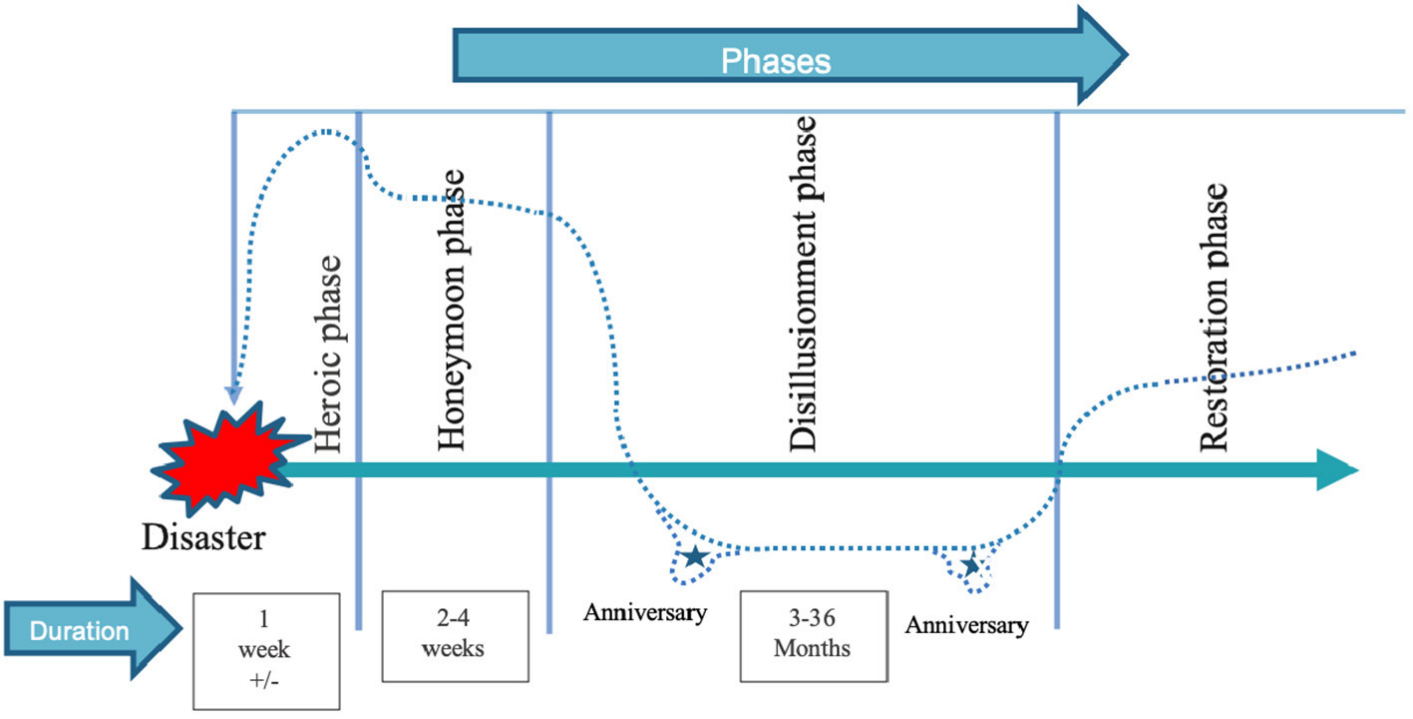
Biopsychosocial response pattern and temporal phases of disaster from Young et al. (34) modified by Math et al. (31, 35).

As the impacts of a natural disaster appear to persist for several years, there is a need to better document the long-term biopsychosocial consequences (33). The *Biopsychosocial response pattern and temporal phases of disaster* (Figure 1) first developed by Young et al. (34) and later modified by Math et al. (31, 35) describes the different phases of expected reactions in individuals experiencing a disaster. According to this model, a “heroic phase” occurs right after the event, can last for a few days, and is described by rescuing, sheltering, feeding and supporting behaviors. Then, a “honeymoon phase”, lasting 2 to 4 weeks ensues, encompasses a period during which individuals are safe, taken care of, and receive help from different organizations that help to provide them a feeling of hope. Several positive effects identified in the P-RAM model such as community cohesion, altruism and adaptive coping probably occur during this phase. Then, a “disillusionment phase” can last from three months to three years. It is a phase of reconstruction and rehabilitation during which the assistance provided to the community and the media attention have diminished. The mental health of the community is more fragile during this phase. Finally, the “restoration phase”, which begins after three years, is described as the gradual return to normal functioning. This model suggests that the consequences of a disaster will evolve and persist over the years. The “disillusionment phase” appears to be a particularly critical period for the affected community and as such, merits more scientific attention.

Few studies have adopted a longitudinal lens to examine the evolution of the consequences of disasters. One of the few longitudinal studies of individuals exposed to wildfires investigated the psychological impacts of the disaster three to four years after the fires, then five years later after the Victorian Black Saturday bushfires in Australia (33). Rates of PTSD, MDD, serious mental illness and problem alcohol use decreased overall over time. However, these rates remained higher than in the general population. Such a quantitative portrait is essential, but having access to individuals' subjective experience and perceptions of the long-term impacts of disasters could help inform mental health professionals' practice even further, for example by documenting important perceptions, attitudes, reactions and main concerns of this particular population (36).

In sum, the existing empirical evidence concerning the consequences of natural disasters has largely explored psychological consequences, while little emphasis placed on other types of consequences presented in the P-RAM model (physical, social, positive effects), leaving significant knowledge gaps. There are also few studies on the long-term consequences of natural disasters, in particular very few qualitative studies, which could help gain a better understanding of the evolution of perceived consequences. Therefore, the overarching aim of the present study was to document subjective experience of wildfire evacuees and the perceived consequences of this type of disaster and how these perceptions evolved over time. More specifically, the first objective of the present study was to document the evacuation experience from the standpoint of the evacuees of the Fort McMurray wildfires. The second objective was to document the biopsychosocial consequences of wildfires as perceived by evacuees from Fort McMurray, three months and three years later.

## Materials and Methods

### Participants and Procedure: Experience of the Fires and Evacuation and Biopsychosocial Consequences 3 Months After the Fires

The data of the present study were collected as part of a larger project including quantitative measures of mental health published elsewhere (18). The Laval University institutional review board approved the research protocol, and participants provided informed consent. Two doctoral students in psychology went to Fort McMurray to recruit participants 3 months after the evacuation (July 25 to August 16, 2016). They distributed invitations to participate in the study to citizens in public places such as retail stores and supermarkets in Fort McMurray. They also publicized recruitment on local radio programs. The participants were invited to complete an online questionnaire. The survey was open between July 25 to September 5, 2016. To be eligible to participate in the study, all respondents had to: (a) be at least 18 years old; (b) have a functional knowledge of English, and (c) have experienced the evacuation from Fort McMurray. No exclusion criteria were applied. A total of 394 participants completed the survey.

At this first time point, in addition to completing validated quantitative questionnaires assessing psychological symptoms for the larger project, participants answered the following open-ended questions: (1) Describe briefly what happened. How did you personally experience the fires? and (2) What consequences have you suffered from the fires? Participants had unlimited typing space to answer.

### Participants and Procedure: Experience of the Fires and Evacuation and Biopsychosocial Consequences 3 Years After the Fires

An e-mail was sent between June and September 2019 to the 141 participants (out of 394) who agreed to be contacted again after completing the first part of the study. They were invited to participate in a semi-structured interview conducted by telephone concerning their experience of the fires and evacuation three years after the event. No financial compensation was offered for participation. Thirty-one (31) individuals agreed to complete the interview and provided separate informed consent. These interviews were conducted by the first author of this study, a graduate student in psychology, and lasted approximately 15 minutes on average. The interviews were audiotaped and then transcribed verbatim by an undergraduate student.

During the interview, participants answered 11 open-ended questions: (1) What consequences of the fires and evacuation are still present in your life? (2) Do you experience psychological consequences? (3) Do you experience physical consequences? (4) Do you experience social consequences? (5) Do you experience financial consequences? (6) Do you experience positive consequences? (7) What did you find helpful during the evacuation? (8) What did you find difficult? (9) If you have had had the choice to evacuate or not, what would you have done? Why? (10) Have you received professional help to cope with the negative consequences of this event? If yes, describe this help. (11) What kind of help do you feel you would like to have today? These more directive questions were formulated in order to access to the full range of biopsychosocial consequences based on the P-RAM model.

### Data Analysis

A qualitative thematic analysis was performed by the first author in order to categorize the participants' responses into the various aspects and consequences of the wildfires and the evacuation experience. The thematic approach based on the work of Paillé and Muchielli (37) was used as it allows the categorization of participant's answers into themes and sub-themes. The P-RAM model provided a framework for synthesizing the results of the present study, but the analysis was not restricted to the categories generated by this model. The coding method was inductive in order to highlight significant themes and sub-themes among the participants' responses (38). Responses from the questionnaires completed 3 months after the fires were first coded. A sample of the data corpus (about the 50 first questionnaires) were used to create the initial codes. When approximately 70 codes were created, these were then transformed into themes and sub-themes. A first draft of the codebook was created by the first author and reviewed throughout the process. The final codebook was then used to code the entire data corpus. This same codebook was used to code the interview data collected three years later. It was, however, modified again to adapt it to the participants' responses at this second stage, resulting in a second codebook.

An inter-judge agreement was reached to ensure the validity of the categorization. Using the codebooks for each assessment time, an undergraduate psychology student carried out an independent coding of 20% of the data corpus (from 78 questionnaires and from 6 interviews). All discrepancies were discussed, and decisions were made based on mutual agreement. The two codebooks were revised and merged into one through inter-judge discussions. The data corpus re- coded a final time by the first author to ensure that codebook adjustments over time were applied systematically to the entire set of results. The QDA miner 4 software (Provalis Research, Montreal) was used to perform the qualitative analysis.

## Results

The results are presented hereafter in four sections. First, the characteristics of the sample are presented. The second section presents results concerning the evacuation experience. Themes are presented in a loose chronological order, from the beginning of the evacuation day up to the phase of relocation. The third section describes the biopsychosocial consequences experienced by the participants three months and three years after the evacuation. Quotes are used to illustrate the themes. Preceding each quote, the gender (“F” for female and “M” for male) and ages of the respondents are indicated. Given the large number of participants in the study, the fourth section presents a frequency analysis quantifying the frequency of occurrence of themes and sub-themes.

### Sample Characteristics

Sociodemographic characteristics of the sample are presented in Table 1. Among the 394 participants who responded to the questionnaires three months after the fires, it was found that one participant was under the age of 18. This participant was excluded from the analyses. The final sample three months after the fire of the study was composed of 291 women and 72 men. The majority of participants were aged between 18 and 34 years (38,7%) and were employed (79,9%). The majority were married or in a common-law relationship (75,5%).

**Table 1:** Sociodemographic characteristics of participants.

**Variables**	**3 months after the fires** **(*n =* 393)**	**3 years after the fires** **(*n =* 31)**
**Gender**		
Male	79 (20,1%)	8 (25,8%)
Female	303 (77,1%)	23 (74,2%)
No answer	11 (2,8%)	
**Age (years)**		
18-34	151 (38,4%)	9 (29%)
35-49	130 (33,1%)	15 (48,4%)
50-64	95 (24,2%)	5 (16,1%)
65+	8 (2%)	2 (6,5%)
No answer	9 (2,3%)	
**Ethnicity**	**X**	
Canadian or European		29 (93,6%)
Hispanic		1 (3,2%)
Membership in a First Nation		1 (3,2%)
**Employment status**		
Employed	302 (76,8%)	23 (74,2%)
Unemployed	27 (6,9%)	8 (25,8%)
Student	7 (1,8%)	
Other	57 (14,5%)	
**Relationship status**		
Married/common-law	283 (72%)	20 (64,5%)
Separated/divorced/widowed	29 (7,4%)	2 (6,5%)
Single	69 (17,6%)	9 (29%)
No answer	12 (3%)	
**Place of living after the fires**	**X**	
In Fort McMurray		22 (71%)
Outside of Fort McMurray		9 (29%)

The sub-sample assessed three years after the fire was composed of 23 women and 8 men. It was discovered after the interview that one participant had not experienced the evacuation. This participant's data are included in the 3-month questionnaire data, because there was no way to identify these data retrospectively, but not in the 3-year interview data. Most participants were aged between 35 and 49 years (48,4%). Twenty-nine identified as Canadian or European (93,6%), one as Hispanic (3,2%) and one as a member of a First Nation (3,2%). As a reference point, in 2016, in the general population of Fort McMurray, 77% identified as Canadian/European origins, about 1,6% as Hispanics and 5,3% as members of a First Nation (39). The majority of participants were employed (74,2%) and married or in a common-law relationship (64,5%). Among these participants, 22 still resided in Fort McMurray, whereas 9 had moved out of town.

### Evacuation Experience of the Fort McMurray Residents

Among the responses describing the participants' evacuation experience, eight themes were identified: preparation for evacuation, the perceived traumatic nature of the evacuation, problems encountered while on the move, assistance received and provided, physical discomfort, conditions, relocation and no problem/no response (Table 2).

**Table 2:** Evacuation experiences.

**Evacuation experience**							
^*^Preparation for evacuation ^*^Evacuated before the mandatory evacuation Prepared in advance in case No time to prepare Got personal belongings at home Picked-up children/animals	^*^The perceived traumatic nature of the evacuation ^*^Traumatic ^*^Chaotic ^*^Stressful ^*^Unpredictable	^*^Problems encountered while on the move Driving threw/near the flames Traffic ^*^Road closed Out of gas/abandoned vehicules ^*^Being separated from friends and family ^*^Communication issues	^*^Assistance received and provided ^*^Helped others/help received from others ^*^No help First responders	**Vulnerability** **conditions** Being hospitalized Not knowing how to drive On foot Being in labor	**Physical discomfort** Eyes burning Breathing problems Asthma Injury	**Relocation** Permanent Temporary	**No problem/no response** Did not encounter any problems or did not answer the question

* *The asterisk indicates themes and sub-themes that emerged in the participants' verbatims at the 3-month and 3-year collect*.

### Preparation for Evacuation

Three months after the evacuation, one theme concerned the efforts made by residents to prepare themselves for the evacuation. Some respondents decided to leave their home before the mandatory evacuation was announced. Some had prepared themselves in advance just in case they would need to evacuate, so they were ready to leave the day of the mandatory evacuation. For example, one participant (F, 36) explained*: “We had already packed valuables and our child's necessities the day prior, just in case”*. Other participants mentioned that they had no time to prepare themselves because the fire was already too close, and they could not access their house. Another respondent (F, 36) wrote: “*By the time we reached our neighborhood it was under a mandatory evacuation as well, so we were not able to go home to retrieve items”*. Others had had the time to access their home to get their personal belongings and pack their cars. They described having to pick up their children (at home, school, nursery) and pets before evacuating the city. For example, one participant (F, 34) wrote: “*I ran to the school to get my children and to the vet to get my cat and then drove out of town to save our lives”*.

Three years after the event, the respondents who had evacuated before the mandatory evacuation reported that it was a helpful factor. One participant (F, 36) said: “*I think my experience is very different from most, because I evacuated early and I went north, so I didn't have this fear that a lot of people did”*.

### The Perceived Traumatic Nature of the Evacuation

This theme referred to the participants' perceptions of the evacuation experience. Three months after the evacuation, one recurrent perception was the unpredictable nature of the evacuation. Participants mentioned that they believed the fire was under control at the beginning of the day. Most people were busy with their usual schedules when the official order came. One participant (F, 23) described: “*The radio was saying that nothing was wrong. On my way to my mom's, I could see flames overtop the trees in the distance of the forest. I dropped my dogs off and was about to head to work when the radio said we had 30 minutes evacuation notice for Beaconhill”*.

Other than the unpredictable nature of the evacuation, three months post-evacuation many participants qualified their experience as traumatic, some as “*the most traumatic experience of* [*their*] *life”*. A related perception was that the evacuation was a chaotic experience. For instance, one participant (F, 46) wrote: “*Then, vehicles started racing through our street and blowing horns, yelling, telling everyone to get out. I ran outside it sounded like a war zone, propane tanks exploding. Dark. Scary”*. Participants also wrote that it was a highly stressful moment. For example, one (F, 19) described: “*The stress was crazy. I had to pack up everything for me and my fiancée, my cat, my parent's dog and wait for my fiancée's mom to come and get me while every person I knew was calling me at the same time”*.

These perceptions of the event as traumatic, chaotic or highly stressful were also mentioned three years later. One respondent (F, 69) expressed: “*There's no words that describes what happened, what we felt, what we went through. It was traumatic, scary, everlasting. Certainly, a learning experience. I don't know, I don't want to use the word hateful, but it got us feeling what we didn't know we were even able to have. It's been three years and I still cannot describe what the fire did to us”*.

### Problems Encountered While on the Move

The participants evoked the conditions in which the evacuation unfolded. In the questionnaire, some respondents reported driving near or through the flames. One participant (F, 33) wrote: “*I drove through the smoke and flames, through all the abandoned vehicles and saw houses on fire”*. Likewise, some had to abandon their vehicle to escape the flames and smoke. In addition to the fire that was approaching on the road, participants pointed out the important traffic congestion they had to face to be able to leave town. The authorities were also forced to close one of the two main roads for a certain amount of time. One participant (F, 25) described: “*the entire town was on a mandatory evacuation and traffic was grid locked. We were told that we were no longer allowed to go south toward Edmonton and that we had to go north and stay in camps (…). It took us almost 5 hours to get there when normally it would take 45 minutes”*. Some evacuees ran out of gas. They wrote about being given gas by strangers, finding gas stations still open or having to find another means of transportation to leave town.

In this community, residents are aware of annual wildfires occurring in the vicinities, but these fires had never reached town in the past. When the evacuation order was given, the population was scattered across town. Respondents wrote about being separated from friends and family during the evacuation. For example, one respondent (F, 32) wrote: “*I had a friend picking up my daughter from school and evacuated with her, so we separated for 24 hours”*. Another participant (F, 49) wrote: “*My husband and I got separated as he made it south and I had to go north”*. In addition to being separated from loved ones, participants reported communication issues because of dead phone batteries, poor network, overloaded cell towers, etc.

In the interviews conducted three years after the disaster, most participants still noted that closed roads, having been separated from friends and family, as well as communications issues, were some of the most difficult aspects of their evacuation experience.

### Assistance Received and Provided

Three months after the evacuation, respondents indicated that during the evacuation, they helped other people to evacuate (e.g., children at school, coworkers, general population) and checked in on neighbors. For instance, one participant (F, 62) explained: “*All along the street, neighbors were checking in with each other, offering help and advice and exchanging contact information, discussing how to contact neighbors who had not come home yet”*. Participants reported receiving assistance from other community members. One participant (M, 55) wrote: “*I had two families helping me packing up my vehicle and one of the couples split up, so they could drive my car to Athabasca* (a small town between Fort McMurray and Edmonton)”. However, some respondents reported not receiving any assistance during the evacuation. For example, someone (F, 45) wrote: “*We fled with flames on both sides of us and no firefighters, police or anyone helping us. They let our neighborhood burn for days and didn't even attempt to fight it”*. There were also first responders among the participants, such as social workers, firefighters and evacuation center workers. One firefighter (M, 32) wrote: “*I was actively involved in the firefight. I had to watch my lieutenant's house and friends' homes burn while I was trying to stop the fire from spreading”*.

Three years later, respondents reported that they appreciated the assistance received during the evacuation. Indeed, participants mentioned organizations that helped them, such as the Red Cross, and people from the community helping each other during the evacuation. However, some participants also perceived the lack of assistance as a negative part of the evacuation. Participants mentioned experiencing delays in receiving assistance from the government and other organizations.

### Vulnerability Conditions

This theme refers to participants who reported situations of extreme vulnerability and were left to fend for themselves. In the questionnaire, one hospitalized participant (M, 49) described: “*I left the hospital one hour after being evacuated. I walked 5km to Thickwood on ramp to be detained by RCMP, left at the airport with no food or water, slept on floor at bottom of escalator and in a wheelchair, an IV needle still in my arm”*. One person (M, 55) reported having to evacuate the city on foot. Another (F, 36) mentioned not being able to drive. One woman (F, 38) was pregnant and wrote: “*I was also 38 weeks pregnant at the time we left. We evacuated north to a work camp where I went into labor and had to be flown to Edmonton and later Calgary to have our son”*.

### Physical Discomfort

During both data collection periods, some people reported experiencing physical discomfort during the evacuation because of the fire and the heavy smoke. Some participants reported having burning eyes, breathing problems and asthma attacks during the evacuation.

### Relocation

This theme describes the temporary or permanent relocation of the evacuees right after the evacuation. Three months post evacuation, participants described moving temporarily to shelters including crisis centers, family and friends' places, hotel/motel rooms or rented lodgings. The duration of the relocation varied from a few days to a few months, and some evacuees had to relocate several times. For instance, one participant (M, 55) wrote: “*We stayed in a work camp overnight and we were flown out on May 4 to Edmonton. We spent nine days in a hotel room and six more weeks in an apartment”*. Respondents also wrote about deciding to relocate permanently to another city.

### No Problem/No Response

Finally, some participants reported having had no problem during the evacuation. Some respondents were in an area unaffected by the fires. In addition, 26 respondents to the online survey left the open-ended questions unanswered.

### Biopsychosocial Consequences

When asked to describe the consequences of the wildfires at three months and three years, participants mentioned negative and positive consequences (Table 3). The negative consequences were grouped under seven themes: material and financial loss, emotional/mental health disorders, cognitive impairments, behavioral changes, spiritual/existential reflections, alterations in social functioning, and physical conditions. Positive consequences were grouped under four themes: posttraumatic growth, resilience/no consequence, altruism and community cohesion.

**Table 3:** Biopsychosocial consequences of the wildfires.

**Negative consequences**							^*^Positive consequences
^*^Material/financial loss ^*^Loss of house and sentimental belongings ^*^Job problems ^*^Financial issues	^*^Emotional/Mental health disorders ^*^Anxiety ^*^Hostility ^*^Guilt ^*^Sadness ^*^Mood fluctuations ^*^Emotional distress after exposure to traumatic reminders ^*^Self-reported mental health disorders (PTSD, anxiety, depression)	^*^Cognitive impair- **ments** Memory problems Poor concentration Impaired decision- making ability Nightmares Flashbacks ^*^Altered perception of safety/hypervigilance	**Behavioral changes** Increase use of tobacco and/or alcohol	^*^Spiritual/ **existential reflections** ^*^Changes in life perception ^*^Uncertainty about the future	^*^Social alterations ^*^Separation Conflicts ^*^Withdrawal ^†^Social changes ^†^Feeling misunderstood	^*^Physical conditions ^*^Changes in physical conditions ^*^Health problems	^*^Resilience/no consequences ^*^Altruism ^*^Posttraumatic growth ^†^Community cohesion

* *The asterisk indicates themes and sub-themes that emerged in the participants' verbatims at the 3-month and 3-year collect*.

*The symbol †: *indicates themes and sub-themes only found during the interviews conducted at the 3-year follow-up*.

### Negative Consequences

#### Material and Financial Loss

One theme included the direct and indirect impacts of the wildfire on participants' material possessions and finances. In the 3-month questionnaire, participants wrote about the loss of their homes and of all their personal belongings. For example, one respondent (F, 58) wrote: “*We lost everything but the clothes we were wearing”*. Financial issues sometimes included difficulties with their insurance company to rebuild their houses. Other financial problems were due to a period of time during which some participants could not work after the evacuation.

At the 3-year follow-up, respondents reported having lost their homes and personal belongings and still reported issues regarding insurance problems, rebuilding delays and job changes.

#### Emotional/Mental Health Disorders

This theme encompasses the range emotions and psychological disorders that participants mentioned when describing their experience. After three months, emotions could be categorized into anxiety, irritability/frustration, guilt and sadness. The anxiety category included fear and stress regarding their experience. Some were stressed or were afraid for their children, their finances, their return to the city, the separation from their family. Some feared other eventual disasters. One participant (F, 33) expressed: “*I am constantly thinking and stressing over things. It's so hard”*. Another category of emotions that participants described was irritability/frustration. Many participants reported feeling frustrated by the decisions made by authorities before and during the evacuation and by not being able to return to Fort McMurray for some time after the fires, whereas others did not identify a specific cause of their emotions. For example, one participant (F, 23) expressed: “*I am a lot more irritable, and easily angered”*. Other emotions reported can be categorized as guilt. Participants reported feeling guilty when comparing their losses to that of others. For instance, one participant (F, 47) explained: “*We were lucky we have our home still and our daughter has hers. But friends of mine don't and they don't know when they will rebuild. I feel very guilty about this. How did I get so lucky when others didn't?”* Some felt guilty about leaving their pets behind. A last category of emotions reported was sadness. Participants mentioned feeling sad about the changes in the city and hearing other people's stories. One participant (F, 37) expressed: “*I felt (…) sadness upon returning and seeing the devastation. Continue to feel sadness to hear stories of people affected”*. Participants reported emotional fluctuations, or that their emotions felt irrational. One respondent (M, 38) described his emotions by an “*emotional roller coaster of feelings”*. In addition, participants mentioned experiencing emotional distress when exposed to traumatic reminders. For instance, participants felt anxious, upset, or afraid when they were exposed to sirens, smoke, fire pits, the smell of fire, pictures representing the event, heavy traffic, etc. There were also participants who specified suffering from a mental health disorder as a consequence of the wildfires. Participants wrote about suffering from PTSD, anxiety and depression.

Three years later, respondents reported the same categories of emotions, including experiencing emotional distress when exposed to traumatic reminders. Some participants also reported still suffering from PTSD, depression and anxiety regarding to the fires.

#### Cognitive Impairments

This category of consequences refers to cognitive alterations and cognitive intrusions reported by participants. Three months after the fires, some respondents had memory lapses about the event (such as being unable to remember some important moments of the evacuation). One respondent (F, 58) explained: “*The next morning my daughter said, “Imagine mom, we drove through that fire yesterday.” but I couldn't remember doing so, even though I know I did. She showed me pics of it, but I am still unable to remember doing so. I do remember seeing embers while driving and was afraid that they would come down on the car and blow the car up. I remember hearing explosions, which later found out were propane tanks”*. Some respondents reported suffering from poor concentration, or impaired decision-making abilities, but they provided few additional details.

Three months after the wildfires, some mentioned having flashbacks during the day and nightmares during their sleep concerning the fires and evacuation. Participants also reported an altered perception of safety and a state of hypervigilance. For instance, one respondent expressed (F, 33): “*I'm feeling overcautious, sometimes I plan for potential emergencies”*.

Three years after the wildfires, among the different types of cognitive impairment, only hypervigilance was highlighted from the interviews. Some participants were still alert to signs of a possible danger. One participant (M, 32) explained: “*Every time I leave my place, I kind of take a look around and do a mental inventory of everything that's there. Just in case something happens while I'm gone”*.

#### Behavioral Changes

This theme includes behavioral changes reported by the evacuees three months after the evacuation. Increased use of tobacco or alcohol highlighted in the answers. Some participants began to smoke again and drank alcohol more frequently compared to before the event. Participants reported very few details about their consumption habits. No participant reported substance abuse or dependence problem three years after the fires.

#### Spiritual/Existential Reflections

Three months after the event, existential thoughts were identified from the answers concerning changes in identity and perception of life. Participants mentioned having a different perception of themselves. For example, one participant (M, 33) wrote: “*My life will never be the same for us. It definitely alters who you are”*. Furthermore, participants felt uncertain about the future. They reported not knowing what they would become in a few months, where they would be, or what they would be doing. For instance, one respondent expressed (F, 19): “*The hardest part of everything was the uncertainty. When would we come back? Would we come back? Would we go back?”*

Three years after the disaster, participants reported having a new outlook on life. For instance, one respondent (M, 49) explained: “*The whole life changed. Who we are, how we identified, all our history and our past is all gone and we're starting over again”*. Participants also remembered the uncertainty. One participant (F, 29) expressed: “*I thought the uncertainty, the fear, leaving your house when you don't know if you're going back, was difficult. I wasn't able to take many belongings with me so there is a loss of security”*.

#### Social Alterations

This theme included the negative impacts on the participants' social life. Three months after the disaster, respondents were preoccupied by their separation from friends and family members after the fires and evacuation. They were concerned about temporary separation due to relocation of the community and about some residents' decision to move from Fort McMurray permanently. For instance, one participant (F, 20) mentioned: “*I got to see my parents every day, my brothers. Now my parents and brothers live 14 hours away in British Columbia”*. Moreover, respondents described feeling more lonely or isolated after the event, or having voluntarily decided to withdraw from certain relationships for personal reasons. For instance, one participant (F, 37) reported: “*withdrawing from certain friends because of their negative outlook/attitude”*. Respondents also experienced conflict in their relationships. Some felt that their relationships were more strained or falling apart. For example, one participant (F, 26) explained: “*My relationship is over. After eight years we couldn't handle the stress and started fighting so much that the relationship was no longer viable or healthy for either of us”*.

Three years later, participants still mentioned the negative impact of the relocation of family members and friends on their social life. They also expressed feeling more isolated than before the fire. Respondents mentioned changes in their relationships. Some reported having a different circle of friends because of relocation or change of employment. Others felt misunderstood by friends or the rest of the community. For instance, one person (M, 42) said: “*I realize that we don't have friends. The people misunderstood that at the time we wouldn't ask for money. I never asked for money. I just, sometimes, you need a person to talk, you know”*.

#### Physical Conditions

This theme included any physical consequences due to the fire and the evacuation reported by participants. In the 3-month questionnaire, some respondents mentioned changes in their physical condition, without providing further details. Some reported sleep problems, changes in appetite, tiredness, exhaustion, and lack of energy. Participants mentioned health problems, such as lung infections, somatic complaints, headaches, high blood pressure, chest congestion, coughing, sneezing, nausea and hypothyroidism.

In the interviews conducted three years after the disaster, participants reported weight changes, sleep problems, and diabetes as physical consequences of the fires and evacuation.

### Positive Consequences

This section encompasses all of the positive consequences that participants described throughout the study. It is divided into four themes: posttraumatic growth, resilience/no consequence, altruism, and community cohesion.

#### Posttraumatic Growth

This theme refers to personal development experienced by residents following the disaster. For instance, one participant the three-month questionnaire (M, 39) explained: “*I lost many opportunities but found new challenges”*. Another person (F, 34) expressed: “*I'm also more grateful for every moment with my family”*.

Throughout the interviews conducted three years later, participants still experienced positive consequences. For example, one respondent (M, 35) said: “*I mean, after going from being very close to suicidal to now… I would say I experience a lot of posttraumatic growth and I am definitely a stronger person than I ever was before”*.

#### Resilience/No Consequences

This theme describes participants who reported little or no consequences of the wildfires. In the 3-month questionnaire, these respondents expressed feeling resilient to the event. For instance, one person (F, 27) explained: “*It wasn't too bad though, I focused on the positives, so I wouldn't say I was that affected by it”*. Some participants also reported not suffering from any consequences of the fires and evacuation. Others simply did not answer the question, possibly meaning that they experienced no consequences or that they did not wish to answer the question. The adoption of new adaptive coping strategies was reported by some participants. For example, one participant stopped smoking after 37 years. Some respondents mentioned focusing on healthy living habits to help deal with various stressors. Others mentioned working on interpersonal communication issues.

Three years after the disaster, respondents still showed resilience or the use of adaptive coping strategies. For instance, one participant (M, 42) explained: “*I tried to get all the positive from all the situations that we have been through. I would say that I learned who I can trust. I learned also how I can manage better the resources that I have. I think I became a better person from being resilient”*.

#### Altruism

Altruism refers to participants who used their experience to help other people after the fires and evacuation. One person in the 3-month questionnaire (F, 34) wrote: “*I look for ways to help others. I think it's made me more aware of the suffering of others, and I look for ways to help ease it”*. Three years after the wildfires, some respondents mentioned volunteering for the Red Cross to help people who go through similar situations.

#### Community Cohesion

This theme refers to the perception that relationships are more tightly woven in the community. This positive consequence was only identified in the interviews conducted three years after the fires. One participant (M, 46) explained: “*Positive consequences I think are social and community-based. (…) It's a shared experience as well, so what I find is that everybody that lived here in 2016 has that shared experience and therefore we have something in common which strengthen the relationships and the community. I think that is positive sense of community building”*.

### Frequency Analysis of Themes

A few themes and subthemes were more prominent among the participants' responses. Concerning the evacuation experience, a large proportion of respondents reported struggles to find their children and pets before evacuating the city (95/393). Problems encountered while on the move were also frequently mentioned: participants reported driving through/near the flames (112/393), driving in traffic (99/393), and being separated from friends and family during the evacuation (75/393). Three years later, a large proportion of respondents recalled communication problems (10/31) and the distressing feeling of uncertainty (9/31).

Negative consequences were reported more often than positive ones. The most frequently mentioned ones concerned material and financial loss. Many participants reported losing their homes and possessions (82/393 three months later; 12/31 three years later) and having financial worries (82/393). Emotional/mental health disorders were also frequently identified. Three months after the fires, many reported sufferring from anxiety (56/393) and three years later, still being hypervigilant to reminders of the trauma (11/31). Finally, certain elements reported by the participants concerning the social consequences of the event appeared more strongly three years after the fires. Responders reported the social impact of relocating their families and friends on their social circle (9/31), and finally, greater community cohesion (8/31).

## Discussion

The main objective of this study was to document the experience of individuals who went through the 2016 Fort McMurray wildfires evacuation, as well as the various consequences following the evacuation. The use of open-ended questions helped to gain an understanding of participants' personal experiences. Regarding the evacuation experience, the qualitative analysis highlighted eight themes: preparation for evacuation, the perceived traumatic nature of the evacuation, problems encountered while on move, assistance received and provided, vulnerability conditions, physical discomfort, relocation and no problem/no answer. When asked about the circumstances of the evacuation, respondents mostly described the difficult conditions under which they traveled from their residence to the temporary accommodation site. The analysis also highlighted seven themes summarizing the negative biopsychosocial consequences of the fire and the evacuation: material and financial loss, emotional/mental health disorders, cognitive impairments, behavioral changes, spiritual/existential reflections, social alterations, and physical conditions. Positive consequences were also reported: posttraumatic growth, resilience/no consequences, altruism, and community cohesion. This study thus provides valuable knowledge to increase preparedness for individuals and communities to cope for future evacuations linked to wildfires.

The present study first provided a better understanding of the evacuation process *per se*. Participants underlined an important step of the evacuation: preparedness. Although some participants had prepared themselves in advance, most reported lacking time to prepare before the evacuation became mandatory, which increased stress and uncertainty as they struggled to reunite with their loved ones, retrieve their belongings and arrange transportation. Unfortunately, this appears to be common during evacuations from wildfires. Indeed, a study of two communities affected by a wildfire found that respectively 57 and 82% of their participants had not had time to prepare to evacuate (27). Another study highlighted the lack of public awareness of the real danger of the disaster (40). The results of the study demonstrate the importance of keeping the public well informed throughout the course of relatively predictable natural disasters, such as wildfires, hurricanes and floods, and of encouraging people to take action by preparing themselves for an eventual evacuation, even when the event seems under control.

When the disaster becomes a real and imminent danger and the evacuation of the population is ordered, there appear to be shared challenges and concerns. At both measurement times, respondents emphasized the heavy traffic and closed roads, as well as separation from relatives. These results are supported by the few studies on the evacuation process due to a natural disaster. Indeed, Wadsworth et al. (41) studied evacuees from Hurricane Katrina. The victims faced traffic for several hours, which was a stressful aspect of the evacuation. Other studies had also observed that sudden and unexpected separation from family members was a major concern during the evacuation from Hurricane Katrina (40), and that the lack of preparedness in addition to traffic increased the distress in Fort McMurray evacuees (42). Another important issue evoked by participants interviewed three years after the fires were communication problems among themselves and with the government during the evacuation. Communication seems to be important as soon as a wildfire strikes, in order to prepare the population for a possible evacuation. Communication from authorities also should continue during the evacuation, in order to keep the population informed of the latest developments, for example, about the location and intensity of fires and road conditions during the evacuation. These elements (traffic, being separated from relatives, communication issues), which were expressed by several participants, seem to represent the core obstacles encountered during the evacuation. These elements were prominent not only immediately after the disaster but also three years later, suggesting that they had a significant impact on evacuees over time. This information provides insight into the process of evacuating a wildfire itself and could guide efforts to improve preparedness of individuals and communities in regions prone to wildfires (e.g. having a family communication plan in case of evacuation, planning/identification of evacuation routes, practice drills).

During the evacuation, respondents expressed that they perceived the fires and the evacuation as a frightening, unpredictable, stressful and traumatic event. For many respondents, it was perceived as a traumatic event that led to many negative consequences. The Fort McMurray evacuation indeed unfolded quickly while the danger was imminent, and the dangerousness of the situation probably increased its chaotic and stressful character (16). It is striking that, three years later, even though very few people were injured in the evacuation, respondents still described the event with the same perceptions. Thus, even when an evacuation goes relatively well, the perception of danger during the traumatic event, more than the actual harm to one's physical integrity, seems to color people's experience. The data show that although evacuation is a life-saving measure, it is a potentially traumatic event in itself.

The present study also documented the experience of individuals who were more vulnerable and dependent upon others during the evacuation process. Clearly, there is a challenge in assisting people in special or vulnerable situations in the midst of a general evacuation. To our knowledge, few studies have considered these persons who may experience the evacuation very differently, compared to the rest of the population. Tally and colleagues (43) studied the impacts of the 2007 San Diego 2007 wildfires on persons receiving services in public mental health clinics in California. They found that compared to non-evacuated counterpart, evacuated persons reported stronger emotional impacts of the fires, confusion about whether to evacuate, and difficulty in obtaining medications. According to these authors, while the rest of the affected population is experiencing a great loss of control, individuals presenting mental health problems or illnesses may be more even more affected by this sense of loss of control (43). In order to better prepare for future events, there is clear need to document further the experiences of more vulnerable individuals during and after disasters, not only persons with mental health issues but also persons with health issues, mobility issues, cognitive impairment or other types of problems.

The second objective of this study was to document the biopsychosocial consequences of Fort the McMurray wildfires using part of the P-RAM model as a guide. Our results revealed elements relating to the behavioral, emotional, cognitive, social, physical, and spiritual consequences described in the model, as well as to the psychosocial benefits. The literature mentions that even if each context is unique, there are three main elements linking all types of disasters together: disasters threaten the lives of a large number of people at the same time (44), they affect the resources of a community, and they have secondary consequences, mainly physical and mental (24). These similarities could explain the common factors between the P-RAM model developed in the context of terrorist attacks and our findings collected in evacuees from a wildfire. There are, however, differences between the themes and sub-themes of the P-RAM model and those observed in this study. Firstly, the theme “material and financial loss”, not included in the P-RAM model, was identified from our findings. Indeed, as early as three months and even after three years, participants reported experiencing financial problems related to their insurance, house reconstruction, job loss and loss of material assets. It was one of the themes that stood out the most among the participants' responses. This is not surprising, given that the Fort McMurray was considered Canada's most expensive natural disaster at the time (26). In addition, the community of Fort McMurray was already struggling economically due to the collapse of the oil industry in 2014 (45). It is possible that a large proportion of community members were financially affected, more than once. These results are consistent with those of Kulig et al. (27) who reported that their participants who experienced a wildfire evacuation had experienced financial problems associated with the loss of their homes, insurance, job loss and relocation. These financial concerns could be critical to individuals' ability to recover from such an event (33). Personal and financial losses are also associated with several forms of psychopathology including PTSD (28).

Secondly, the “spiritual” category in the P-RAM model focused primarily on philosophical and religious beliefs. In the present study, no religious references were found in participants' responses. These results differ from those usually found in populations affected by natural disasters, where survivors frequently refer to their religion to give meaning to their lives despite the disaster (46). It is possible that the participants in this study were less religious than the populations formerly studied. We found it more relevant to include a category of “spiritual/existential reflections” to highlight the existential aspect of the participants' questioning of their life and future.

In line with the P-RAM model however, several types of consequences appeared to be directly or indirectly related to normal and abnormal psychological reactions, particularly symptoms of PTSD. For example, the emotional/mental health disorders category included a persistent negative emotional state (anxiety/fear, irritability, guilt, etc.) and distress from exposure to traumatic reminders. Anxiety/fear was very present in participants' responses both three months and three years after the fires. Similarly, some PTSD symptoms, such as difficulty concentrating and memory loss, were included in the cognitive impairments category and were particularly prominent three months after the event. Hypervigilance was apparent three months after the fire and was still present three years later. This constant state of alert could be associated with thoughts such as mistrust, suspicion and negative expectations about the future (47) and may persist over time (48). Moreover, spiritual and existential reflections included different perceptions of oneself and uncertainty about the future. This is quite similar to parts of the DSM definition of PTSD where negative beliefs or expectations about oneself, others, or the world that can be observed (49). Finally, we considered sleep difficulties as physical consequences of the wildfire, yet these are also a common symptom of PTSD (American Psychiatric Association, 2000). All these symptoms associated with different biopsychosocial categories that were identified throughout the participants' discourse demonstrate the great impact of the event on their mental health. From a clinical perspective, the results show the importance of providing support and services to victims of a natural disaster over a long period of time, given that these elements associated with mental illness were reported three months and three years later.

Interestingly, participants' responses showed a positive evolution of social consequences over time. Indeed, three months after the fires, respondents reported more negative consequences (conflict, separation, isolation), whereas three years after the fires, respondents reported more positive changes in their relationships as well as community cohesion. These results are congruent with those of a study by Gibbs et al. (50), which found an increase in sense of community three to four years after wildfires in Australia. Another study of wildfires in the Canadian Northwest Territories also showed that a year later, the fires brought new opportunities for people in the community to help each other and bond together after these events (17). These results are also consistent with the *Biopsychosocial response pattern and temporal phases of disaster*, which describes the disillusionment phase, commencing about three months after the disaster, as a phase during which victims receive less social support. From three years onwards, the restoration phase corresponds to the return of harmony in the community (31). One hypothesis is that initially, the high intensity of short-term stressors (e.g., relocation, loss of property, PTSD symptoms) may affect the quality of some interpersonal relationships and that it is only with some distance, several months or years later, that bonds are rebuilt, or others are formed in the community. These findings provide further support for the potentially positive evolution of the social impact of disaster over time. Further research is needed to understand factors that promote community cohesion and to identify ways to harness the power of community to enhance the mental health of individuals.

Finally, positive consequences such as posttraumatic growth emerged very soon after the disaster and were still present three years later. A study by Tedeschi and Calhoun (51) identified five categories of possible positive types of posttraumatic growth: new possibilities, relating to others, personal strength, spiritual change, and appreciation of life. Many of our participants' responses related to these different categories. Other consequences that had a positive impact on participants were resilience, altruism and community cohesion. Participants reported being able to use their resources appropriately to deal with the aftermath of the disaster, either by making positive changes for themselves or by sharing their time and experience with other evacuees. Some authors posit that such positive actions could contribute to reduce post-traumatic symptoms over time (52). Further research should examine whether recognizing positive consequences of disasters could be incorporated in psychotherapeutic interventions. Indeed, the latter mainly focus on negative consequences, yet mental health professionals could help evacuees develop awareness of positive consequences which might have emerged from a stressful event such as an evacuation, and harness the potential of positive actions and perceptions to enhance recovery.

## Limitations

The results of this study need to be interpreted in light of certain methodological limitations. Firstly, cultural diversity of the sample may have been limited as no information was collected on the ethnicity of participants at the 3-month data collection and a large majority of participants in the 3-year data collection identified as Canadian or European. Secondly, the two data collection time points used different evaluation modalities (questionnaires and interviews). It was therefore not possible to thoroughly compare data from the two measurement time points because this was not a quantitative study. In addition, the questionnaire modality used at the 3-month assessment did not allow for additional questions in the case of incomplete answers. This may have resulted in some responses lacking in detail, such as for physical conditions, cognitive alterations and behavioral changes. However, the use of interviews during the second data collection made it possible to further question participants in order to obtain more in-depth information. The use of different evaluation methods also made it possible to obtain a diversity of responses. A future study could evaluate consequences in a more systematic manner with several measurement times and following more closely the biopsychosocial response pattern and temporal phases of disaster [Young et al. (34) modified by Math et al. (31, 35)] in order to empirically test the implications of this theoretical model.

Despite these limitations, this study also has several strengths. For a qualitative study, the sample size is quite large sample size, with almost 400 participants in the first data collection and over 30 participants. This ensured that excellent data saturation was achieved. The large sample size made it possible, among other things, to detect rare situations involving specific vulnerabilities (e.g., mobility issues, hospitalization, advanced pregnancy). Future qualitative studies should however specifically recruit people in various situations of vulnerability in order to better document their experience and their specific needs during evacuations in order to adapt emergency plans to these individuals.

## Conclusion

In conclusion, this study has brought to light a large scope of biopsychosocial consequences experienced by the victims of the wildfires of Fort McMurray over a period of almost three years and documented their evacuation experience. With regard to evacuation, the present study clearly highlighted its traumatic aspect. Overall, the study found that it is important to pay attention to all biopsychosocial consequences, not only mental health consequences, as they all have an impact on evacuees. In addition, the qualitative approach emphasized the importance of attending to both negative and positive consequences of such a disaster, all of which are part of the participants' experience and can influence interventions with evacuees. Finally, this study underscores that there is a need to intervene and maintain services over the long term given the persistence of the consequences over time.

## Data Availability Statement

The raw data supporting the conclusions of this article will be made available by the authors, without undue reservation.

## Ethics Statement

The studies involving human participants were reviewed and approved by Comités D'éthique de la Recherche avec des êtres humains de l'Université Laval (CÉRUL). The patients/participants provided their written informed consent to participate in this study.

## Author Contributions

GB, M-CO, and CM designed the study. A research team collected the data three months after the fire. LT collected, analyzed, and interpreted the data three years after the fire and wrote the manuscript. All authors revised the manuscript and approved the final version before submission.

## Funding

This research was funded by Institute for Catastrophic Loss Reduction.

## Conflict of Interest

The authors declare that the research was conducted in the absence of any commercial or financial relationships that could be construed as a potential conflict of interest.

## Publisher's Note

All claims expressed in this article are solely those of the authors and do not necessarily represent those of their affiliated organizations, or those of the publisher, the editors and the reviewers. Any product that may be evaluated in this article, or claim that may be made by its manufacturer, is not guaranteed or endorsed by the publisher.
